# Validation of Appropriate Reference Genes for qRT–PCR Normalization in Oat (*Avena sativa* L.) under UV-B and High-Light Stresses

**DOI:** 10.3390/ijms231911187

**Published:** 2022-09-23

**Authors:** Hang Yin, Danni Yin, Mingzhi Zhang, Zhiqiang Gao, Muzhapaer Tuluhong, Xiaoming Li, Jikai Li, Bing Li, Guowen Cui

**Affiliations:** 1 Department of Grassland Science, College of Animal Science and Technology, Northeast Agricultural University, Harbin 150030, China; 2 Institute of Grass Research, Heilongjiang Academy of Agricultural Sciences, Harbin 150080, China

**Keywords:** oat, reference genes, qRT–PCR, UV-B, high-light, stability evaluation

## Abstract

Oat is a food and forage crop species widely cultivated worldwide, and it is also an important forage grass in plateau regions of China, where there is a high level of ultraviolet radiation and sunlight. Screening suitable reference genes for oat under UV-B and high-light stresses is a prerequisite for ensuring the accuracy of real-time quantitative PCR (qRT–PCR) data used in plant adaptation research. In this study, eight candidate reference genes (sulfite oxidase, *SUOX*; victorin binding protein, *VBP*; actin-encoding, *Actin1*; protein PSK SIMULATOR 1-like, *PSKS1*; TATA-binding protein 2-like, *TBP2*; ubiquitin-conjugating enzyme E2, *UBC2*; elongation factor 1-alpha, *EF1-α*; glyceraldehyde-3-phosphate dehydrogenase 1, *GAPDH1*;) were selected based on previous studies and our oat transcriptome data. The expression stability of these reference genes in oat roots, stems, and leaves under UV-B and high-light stresses was first calculated using three frequently used statistical software (geNorm, NormFinder, and BestKeeper), and then the comprehensive stability of these genes was evaluated using RefFinder. The results showed that the most stably expressed reference genes in the roots, stems, and leaves of oat under UV-B stress were *EF1-α*, *TBP2,* and *PSKS1*, respectively; the most stably expressed reference genes in the roots, stems, and leaves under high-light stress were *PSKS1*, *UBC2,* and *PSKS1*, respectively. *PSKS1* was the most stably expressed reference gene in all the samples. The reliability of the selected reference genes was further validated by analysis of the expression of the phenylalanine ammonia-lyase (*PAL*) gene. This study highlights reference genes for accurate quantitative analysis of gene expression in different tissues of oat under UV-B and high-light stresses.

## 1. Introduction

Oat (*Avena sativa* L.) is a dual-purpose food and forage crop species of the family Poaceae in the genus Avena, and is the sixth most grown crop species worldwide, behind that rice, sorghum (*Sorghum bicolor* L.), maize, wheat, and barley (*Hordeum vulgare* L.). Oat is nutritious, the seeds are rich in dietary fiber and β-glucan [1], and the bran is rich in triterpenoid saponins, vitamins B, protein, fats, and minerals [2,3]. In addition, the stems and leaves can also be used as feed for herbivores because of their high forage value [4]. Owing to its cold tolerance, drought tolerance, resistance to soil infertility, and high adaptability, the oat has become a dominant cultivated forage grass species in the Tibetan Plateau region. Due to the high altitude, low air density and high atmospheric transparency associated with plateaus, the direct radiant energy reaching the ground is high, so plants growing on plateaus must tolerate strong UV-B and high-light for long periods of time [5,6]. Understanding gene function and expression in oat in adaption to UV-B and high-light irradiance in alpine areas can lay a theoretical foundation for breeding highly resistant varieties.

Real-time quantitative PCR (qRT–PCR) is a method for real-time monitoring of the fluorescence signal of each cycle of amplification and for quantitatively analyzing the template by amplification during the exponential phase [7]. Owing to its high sensitivity, high specificity, reliable results, and ease of performance, qRT–PCR has been extensively applied for gene expression analysis in the field of molecular biology [8]. However, during actual experimental operation, the quantitative results can be impacted by factors including extraction of RNA, reverse transcription, and qRT–PCR amplification efficiency [9,10]. Therefore, in order to ensure the accuracy of the experimental results, suitable reference genes need to be used as standards to measure the expression level of the target gene. In botany, most of the reference genes encode proteins that function as an essential component of the organism’s cytoskeleton structure (e.g., *ACT*, *TUB*, etc.) and are partially associated with the biochemical metabolic pathways of cells (e.g., *EF1-α, GAPDH*, *UBQ*, etc.); these gene products are essential for cellular biological activity, and they can theoretically be expressed stably under any conditions. The idea reference gene must be expressed at a relatively stable level in different tissues and be unaffected by environmental or biological stresses. However, there is not yet a reference gene that is stably expressed across all tissues and organs or under all stress conditions [11,12]. If reference genes are not selected properly, the results of an experiment may be affected, or even the opposite conclusions may be obtained. Thus, it is important to select the optimal reference genes for different plant materials and tissues, according to the specific experimental requirements to ensure accurate and reliable results.

GeNorm, NormFinder, and BestKeeper are three frequently used statistical software in assessing the stable expression of candidate reference genes and selecting suitable reference genes, which are then analyzed from different perspectives. Meanwhile, RefFinder is a comprehensive analysis tool that is integrated with geNorm, BestKeeper, NormFinder, and the ΔCt methods. By comparing the analysis results of statistical software, researchers can select optimal reference genes. In recent years, these statistical software have been used to successfully identify the optimal reference genes for many species, such as wheat (*Triticum aestivum* L.) [13], Arabidopsis (*Arabidopsis thaliana* L.) [14], rice (*Oryza sativa* L.) [15] maize (*Zea mays* L.) [16] and *Aegilops tauschii* [17]. Although, gene function analysis has been conducted in oat and the verification of optimal reference genes under several abiotic stresses has been reported [18,19]. There are still no detailed studies on the stability of oat candidate reference genes under UV-B and high-light stresses. In the present study, eight candidate reference genes (*SUOX*, *VBP*, *Actin1*, *PSKS1*, *TBP2*, *UBC2*, *EF1-α*, and *GAPDH1*) were chosen based on previous studies and our oat transcriptome data, and their expression stability in the roots, stems, and leaves of oat under UV-B and high-light stresses were assessed by geNorm, NormFinder and BestKeeper, and the comprehensive stability was ranked by RefFinder. Moreover, we measured the expression level of phenylalanine ammonia-lyase *(PAL)* using two most stable and least stable reference genes to validate the reliability of the results. Our findings provide a basis for further study on the expression of genes in plants under UV-B and high-light stresses.

## 2. Results

### 2.1. Analysis of Primer Specificity and Amplification Efficiency

In this study, eight candidate reference genes were selected from our oat transcriptome data, and corresponding PCR primers were designed. Details of these candidate reference genes were shown in Table 1. The primer specificity of the candidate reference genes was verified by PCR and qRT–PCR. cDNA from oat seedling leaves was used as a template for PCR. The results suggested that the size of the amplification products for each reference gene ranged from 104 bp for the *UBC2* primer to 147 bp for the *EF1-α* primer, with only one specific band for each primer, and the fluorescence quantitative melting curve also showed a single amplification peak (Table 1, Figures S1 and S2). The amplification efficiencies of the eight candidate gene primers ranged from 92.61% for *PSKS1* to 109.42% for *UBC2,* and the coefficient of determination (R^2^) varied from 0.9629 (*UBC2*) to 0.9998 (*PSKS1*) (Table 1).

### 2.2. Expression Profiles and Stability of Candidate Reference Genes

**Figure 1:**
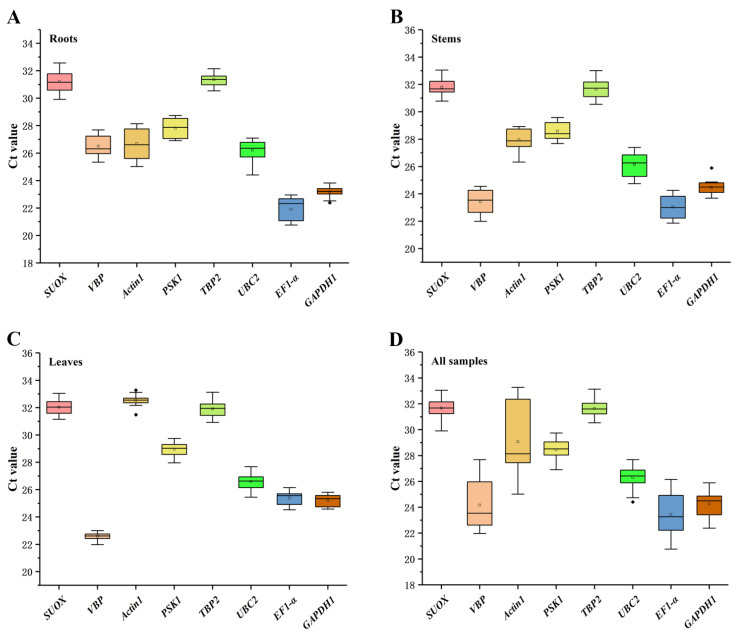
Distribution of Ct values among eight candidate reference genes in the roots (**A**), stems (**B**), leaves (**C**), and all analyzed samples (**D**). Boxes indicate the 25th and 75th percentiles, with the line across the boxes representing the medians. The whiskers and asterisks represent the 95% confidence intervals and outliers, respectively.

As shown in Figure 1, the expression levels of the eight candidate reference genes were presented according to cycle threshold (Ct) values, and the lower Ct values indicated higher gene expression levels. *EF1-α* has the highest expression in roots and stems, and the highest expression gene in leaves is *VBP.* The order of gene expression levels in all samples from high to low is *EF1-α* > *VBP* > *GAPDH1* > *UBC2* > *PSKS1* > *Actin1* > *TBP2* > *SUOX.* The Ct values in the roots, stems, leaves, and all samples ranged from 21.91 to 32.54. *GAPDH1* showed the least variation in the roots and stems (Figure 1A,B), whereas *VBP* showed the least variation in the leaves, suggesting that the most stably expressed gene varied according to the tissues (Figure 1C). In addition, the top two stably expressed candidate reference genes in all the samples were *TBP2* and *PSKS1*, and the least stably expressed gene was *Actin1* (Figure 1D).

### 2.3. GeNorm Analysis

**Figure 2:**
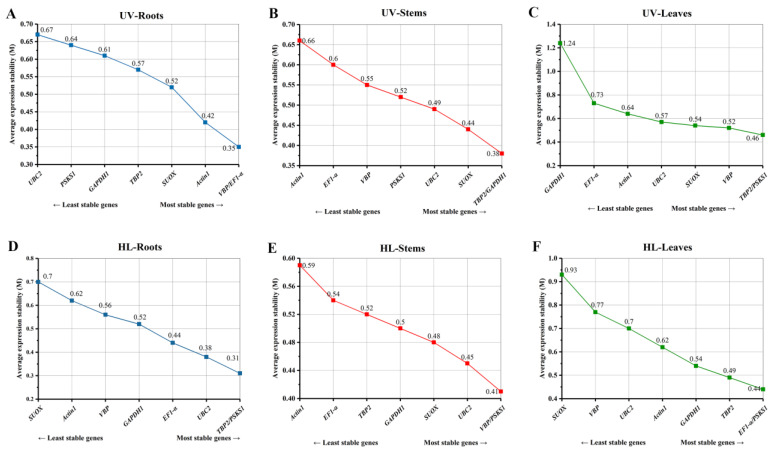
Expression stability of eight reference genes in different oat tissues under UV-B (**A**–**C**) and high-light (**D**–**F**) stresses based on geNorm analysis.

The average expression stability (M) was calculated by geNorm to determine the average expression level of the eight reference genes across different oat tissues under various stresses (Figure 2). Usually, Reference genes with M values below a threshold of 1.5 are considered to be stable, and all the candidate reference genes in this study were stably expressed (M < 1.5). For UV-B stress, the expression of *VBP* and *EF1-α* (M = 0.35), *TBP2* and *GAPDH1* (M = 0.38), and *PSKS1* and *TBP2* (M = 0.46) was the most stable, while *UBC2* (M = 0.67), *Actin1* (M = 0.66) and *GAPDH1* (M = 1.24) was the least stable in the roots, stems and leaves, respectively (Figure 2A–C). In addition, *PSKS1* expression was highly stable in most tissue types under UV-B stresses, while *Actin1* and *SUOX* were usually the least stably expressed genes. Under high-light stress, *PSKS1* and *TBP2* (M = 0.31), *PSKS1* and *VBP* (M = 0.41), and *PSKS1* and *EF1-α* (M = 0.44) were the most stably expressed gene in the roots, stems and leaves, respectively (Figure 2D–F), while SUOX and *Actin1* were the least stably expressed genes.

**Figure 3:**
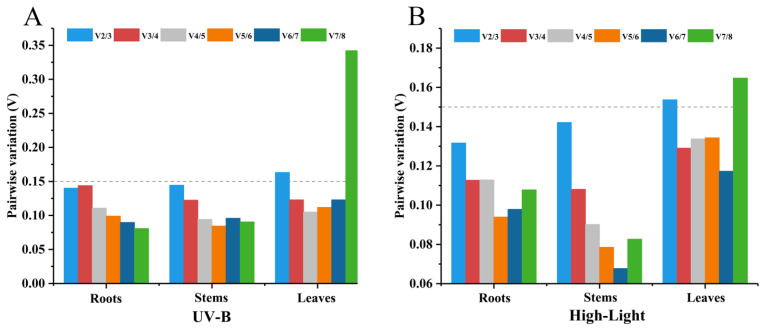
geNorm analysis of the pairwise variation (V) values for eight candidate reference genes under UV-B (**A**) and high-light (**B**) stresses.

In geNorm analysis, the pairwise variant analysis provides criteria for selecting the optimal combination of the number of candidate reference genes, and if the V_n_/V_n + 1_ is less than 0.15, the best solution is the top n reference genes ranked together as the reference genes. As shown in Figure 3, V_3_/V_4_ was less than 0.15 in the leaves under UV-B and high-light stresses, indicating that three reference genes should be used for the normalization of target gene expression data. V_2_/V_3_ was less than 0.15 in the roots and stems under UV-B and high-light stresses, indicating that two candidate reference genes are needed for normalizing gene expression data.

### 2.4. BestKeeper Analysis

The stability of the expression levels calculated by BestKeeper is based on the coefficient of variation (CV) and standard deviation (SD), and the smaller values represent a more stable expression. The rankings showed that the most stable genes were *SUOX* (roots and leaves) and *UBC2* (stems) under UV-B stress, while the least stable gene was *Actin1* (roots and stems) and *GAPDH1* (leaves). Under high-light stress, the most stable expressed gene in the roots, stems, and leaves were *PSKS1*, *UBC2,* and *EF1-α*, respectively. Inversely, *SUOX* was the least stable gene in roots and leaves, and *Actin1* was the least stable in stems. *UBC2* and *PSKS1* were the top two reference genes stably expressed in all samples, while *Actin1* was the least stable expressed gene (Table 2).

### 2.5. NormFinder Analysis

In NormFinder analysis, a lower stability value (SV) indicates greater stability. As shown in Table 3, the most stably expressed candidate reference genes in the roots, stems, and leaves under UV-B stress were *EF1-α*, *TBP2,* and *VBP*, and the least stably expressed genes were *UBC2*, *Actin1,* and *GAPDH1*. Under high-light stress, the most stably expressed gene in the stems was *UBC2*, and the least stably expressed gene was *Actin1*. *PSKS1* was the most stably expressed gene in both the roots and the leaves, and *SUOX* was the most instability expressed gene. Meanwhile, *PSKS1* was the most stably expressed reference gene in all the samples, and *VBP* was the least stably expressed gene.

### 2.6. RefFinder Analysis

Due to the different calculation methods of geNorm, Bestkeeper, and NormFinder, the rankings obtained showed great differences. RefFinder is a comprehensive analysis tool that could provide an overall ranking by assigning an appropriate weight to an individual reference gene and calculating the geometric mean of their weights based on the rankings from geNorm, BestKeeper, NormFinder, and the ΔCt methods. In recent years, it has been widely used for evaluating gene expression stability when the results of geNorm, NormFinder, and BestKeeper were inconsistent. Thus, we finally adopted the RefFinder algorithm to comprehensively rank the expression stability of all the candidate reference genes in different tissues under various stresses. The comprehensive analysis showed that *EF1-α*, *TBP2,* and *PSKS1* were the most stably expressed genes in the roots, stems, and leaves, respectively under UV-B stress. Meanwhile, *PSKS1*, *UBC2,* and *PSKS1* were the most stably expressed genes in the roots, stems, and leaves, respectively under high-light stress (Table 4). *PSKS1* was the most stably expressed candidate reference gene in all samples, and *VBP* was the least stably expressed candidate reference gene.

### 2.7. Reference Gene Validation

**Figure 4:**
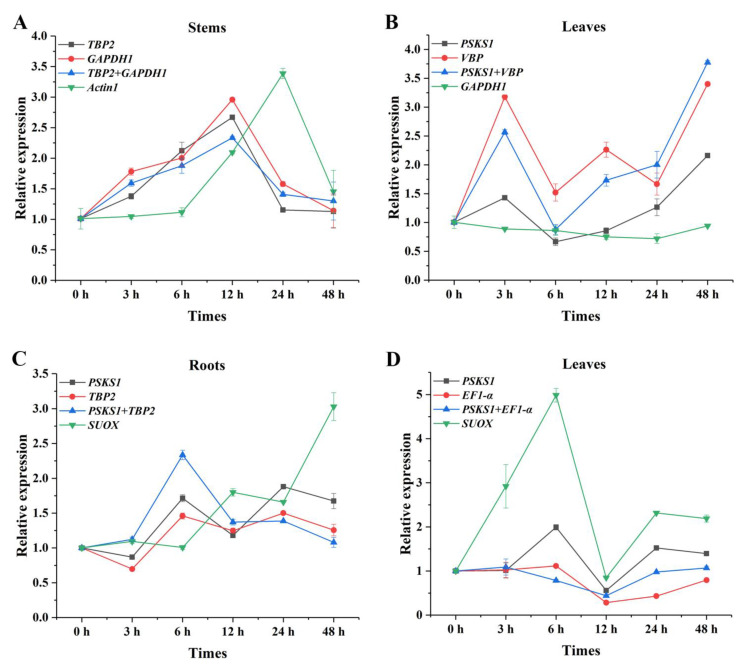
Relative expression level of *PAL* in the stems and leaves under UV-B stress (**A**,**B**) and the roots and leaves under high-light stress (**C**,**D**) when the most and least stably expressed reference genes were used for normalization.

To validate the accuracy of the results of the analysis of expression stability of the reference genes, we analyzed the relative expression of *PAL* genes in randomly selected tissues under UV-B and high-light stresses with two most stably expressed and one unstably expressed candidate reference gene according to the RefFinder results. The expression of *PAL* exhibited similar trends when the most stably expressed reference gene was used alone or in combination either in different tissues or under abiotic stresses; whereas the obtained expression patterns of the most unstably expressed reference genes obviously differed from the optimal reference genes used alone or in combination (Figure 4).

It was evident that the expression patterns of *PAL* were similar in the stems under UV-B stress when *TBP2*, *GAPDH1*, and *TBP2* + *GAPDH1* were used as reference genes, respectively. When the data was normalized using *TBP2*, *GAPDH1*, and *TBP2* + *GAPDH1*, the expression level of *PAL* showed more than doubles at 3 h and 6 h compared with 0 h. However, almost no difference was shown in the expression trend of *Actin1* normalization results at 3 h to 6 h. In addition, the expression level of *PAL* was 3.39 times than that of the 0 h when the unstably expressed gene *Actin1* was used as reference gene, which was much higher than that of the stable reference gene and its combination as the reference gene (Figure 4A). The expression patterns of *PAL* normalized by *PSKS1*, *VBP*, and their combination showed a similar tendency over time in the leaves under UV-B stress, but there was no obvious change when *GAPDH1* was used as the reference gene (Figure 4B). Similar differences were also found under high-light stress (Figure 4C, D). In roots, when unstably expressed reference gene *SUOX* was used as the normalizing gene, the expression of the *PAL* gene showed an opposite decreasing or increasing trend compared with *PSKS1*, *TBP2*, and *PSKS1* + *TBP2* at 6 h and 48 h, respectively (Figure 4C). In leaves, the expression level of *PAL* varied much more when normalized by *SUOX* compared with that of the most stably expressed reference genes (Figure 4D). Inappropriate reference genes may overestimate or underestimate the expression of target genes. These results indicated that the selection of optimal reference genes is extremely important in the study of gene expression.

## 3. Discussion

Gene expression analysis is an important tool to understand biological regulatory mechanisms, and the use of stably expressed reference genes is a precondition to ensure reliable qRT–PCR results because it is highly affected by species, tissues, RNA quality, and other environmental factors [20,21]. Currently, the effect of select reference genes on the accuracy of fluorescence quantitative data has been studied in many plant species, and optimal reference genes have been identified in each of those species [22,23,24,25]. Some genes (e.g., *actin*, *β-actin*, *EF1-α*, *GAPDH*, *CYP,* and *18S*) have been identified as optimal reference genes and they have been widely used in molecular biology research in many species. Studies involving reference gene screening have also been carried out in oat. For example, *EF1-α*, TATA-binding protein, *GAPDH,* and *PGD* can be served as reference genes under salt stress, *ADPR*, and *GAPDH* can be used in the leaves and the roots, respectively, under drought stress, and *ADPR* is also considered to be a suitable reference gene under cold or heats stress [18,19]. However, optimal reference genes may be varied depending on tissues, organs, and stresses, and there is still a lack of studies on the optimal reference genes under UV-B and high-light stresses.

In this study, eight selected reference genes are all highly specific (Figures S1 and S2), and the expression analysis was conducted in three tissues, namely the roots, stems, and leaves. The results of geNorm and NormFinder were generally consistent but were slightly inconsistent with those of BestKeeper, which is related to the computational differences between BestKeeper and the other two methods [26,27,28]. RefFinder is a comprehensive analysis tool that is integrated with geNorm, BestKeeper, NormFinder, and the ΔCt methods. The combined results of RefFinder indicated that *EF1-α* was the most stably expressed gene in the roots of UV-B stressed plants; *EF1-α* is ubiquitous and has a number of important functions. It not only is involved in many important biological activities and disease processes, including translational control, apoptosis, cytoskeleton composition, and signaling, but also is a stably expressed reference gene in Chicory (*Cichorium intybus* L.) and Parsley (*Petroselinum crispum* L.) [29,30]. *TBP2* has a crucial role in transcriptional processes in the nucleus of eukaryotic cells [31,32] and has been shown to serve as a reference gene in papaya (*Carica papaya* L.) under ethylene and as a reference gene in Siberian wild rye (*Elymus sibiricus* L.) adapted to salt stress [33,34]. Our results also showed that *TBP2* can be used as a reference gene in the stems of oat under UV-B stress, and confirmed. *UBC2* was the most stably expressed gene in the stems under high-light stress; The *UBC2* gene is an important component of the ubiquitin/proteasome pathway and has an important role in the ubiquitination of proteins. Due to its highly conservative character, *UBC2* serves as a stably expressed reference gene for different tissues in red clover (*Trifolium pratense* L.) [35], and licorice (*Glycyrrhiza glabra* L.) and for phytohormone and NaCl stress responses [36] in licorice and under drought stress [37]. Furthermore, *PSKS1* was the most stably expressed candidate reference gene in the leaves under UV-B stress and in the roots and leaves under high-light stress. *PSK* is a novel plant peptide growth regulator discovered in recent years that has a very wide range of biological activities and effects. It has been reported that *PSK* genes promote cell division, cell differentiation, and somatic cell formation, coordinate the metabolism and utilization of glucose, help resist oxidative stress, increase chlorophyll contents, improve salt tolerance and high-temperature tolerance of Arabidopsis, and promote the development of pear pollen tubes, and these genes are considered chemical caretakers of plant cell development processes [38,39,40]. A combined analysis of all the samples in the current study indicated that *PSKS1* was the most stably expressed candidate reference gene and that *VBP* was the least stably expressed candidate reference gene.

To verify the reliability of the selected candidate reference genes, we used the top two most stably expressed reference genes, their combinations and the least stably expressed reference genes to normalize the relative expression levels of *PAL* in three tissues of oat. *PAL* is known as the rate-limiting enzyme in the phenylpropanoid pathway [41]. The intermediates produced in the phenylpropanoid pathway can directly or indirectly synthesize a variety of plant secondary metabolites, including flavonoids, lignin, phenol, plant antitoxin, and capsaicin [42,43,44,45]. Phenylpropanoids are widely distributed throughout the plant kingdom, and they constitute a class of secondary metabolites and play essential roles in plant development by acting as essential components of cell walls and protective agents against abiotic stresses [42]. Several studies have demonstrated that UV-B and high-light stress both can change *PAL* expression, thereby affecting plant growth [46,47,48,49]. In this study, the expression patterns of *PAL* were mainly congruent when the top two most stably expressed genes were used as reference genes for normalizing the expression of target gene. Although the expression trend of each individual stably expressed reference gene was consistent with that of the combination of stably expressed reference genes, there were still subtle differences in expression levels. However, the expression of unstable reference genes showed the opposite result, demonstrating that the relative expression of target genes varied with the difference and number of selected reference genes.

## 4. Materials and Methods

### 4.1. Plant and Stress Treatments

The oat (*Avena sativa* L.) variety Qingyin No. 1, provided by the Qinghai Academy of Animal Science and Veterinary Medicine, China. Oat seeds were germinated on filter paper, and then seedlings with a uniform appearance were transferred to seedling trays (soil: vermiculite [1:1], room temperature, 16/8 h light/darkness photoperiod with a light intensity of 300 μmol/m^2^/s) and watered every 2 days with Hoagland nutrient solution. When the seedlings reached the three-leaf stage, they were subjected to abiotic stress by way of UV-B (500 μW/cm^2^) and high-light (1800 μmol/m^2^/s^1^) stresses, and the roots, stems, and leaves were harvested at 0 h, 3 h, 6 h, 12 h, 24 h, and 48 h, respectively. The seedlings of oat were immediately frozen in liquid nitrogen and stored in −80 °C refrigerators. Each experimental treatment was biologically replicated three times.

### 4.2. Total RNA Extraction and cDNA Synthesis

The total RNA of samples was extracted with an Ultrapure RNA Kit (CWBIO, Taizhou, China), a plant RNA extraction kit, following the manufacturer’s instructions. After subjecting the RNA quality to 1% agarose gel electrophoresis (WD-9413B, Liu Yi Biological Technology Co., Ltd., Beijing, China) and ultramicroscopic spectrophotometry (NanoPhotometer N50, IMPLEN, Munich, Germany), the reverse transcription of RNA was carried out with 0.1 μg of total RNA, and a 20 μL reaction mixture using HiScript III 1st Strand cDNA Synthesis Kit (+gDNA wiper) (Vazyme, Nanjing, China).

### 4.3. Selection and Primer Design of Candidate Reference Genes

We selected the five widely used reference genes including *EF1-a*, *TBP2*, *GAPDH1*, *UBC2,* and *Actin1* as candidate reference genes by searching the Internal Control Genes (ICG, http://icg.big.ac.cn/index.php/Species:Plant, accessed on 12 November 2021), a reference gene database, and the relevant literature in sequenced Graminae species [18,50] and obtained their sequences from the NCBI database (https://www.ncbi.nlm.nih.gov, accessed on 16 November 2021). Using TBtools software (https://github.com/CJ-Chen/TBtools, accessed on 18 November 2021), the sequences were used as query files to obtain the sequences of the candidate reference genes by comparison with the transcriptome data sequenced by our research group. Meanwhile, three stably expressed genes (*SUOX*, *VBP* and *PSK*) under different treatments were chosen from our previous transcriptomic dataset. Gene-specific primers of these genes for use in qRT–PCR were designed using Primer Premier 5.0 software (Premier, Inc., Toronto, ON, Canada) (Table 1). The primers were 20–24 bp in length with an annealing temperature of 55–65 °C, a GC content of 45–60%, and product size of 100–150 bp. The primers were synthesized by Rui Bo Xing Ke Biotechnology Co., Ltd. (Beijing, China).

### 4.4. qRT–PCR Analysis of Candidate Reference Genes

cDNA from the roots, stems, and leaves were diluted to 0.1, 0.01, 0.001, and 0.0001 times the initial concentration, and it was used for calculations of the amplification efficiency (E) and the coefficient of determination (R^2^) of the primers and to construct standard curves: E (%) = (10^−1^/slope^−1^) × 100. The cDNA of samples was diluted to approximately 200 ng/μL and used for comparing the stability of different primers in different oat tissues. Three technical replicates were performed for each biological replicate.

In this case, qRT–PCR was performed on the Quantagene q225 system (Kubo, Beijing, China). Each 10 μL reaction mixture contained 5 μL of 2 × ChamQ SYBR Color qPCR Master Mix (High ROX Premixed, Vazyme, Nanjing, China), 0.5 μL of cDNA, 0.4 μL of forward and reverse primer, and 3.7 μL of ddH_2_O. The procedure of the instrument was as follows: 40 cycles of 180 s at 95 °C; 10 s at 95 °C, and 30 s at 58 °C, followed by 72 °C. The melting curve was automatically generated at the end of each run.

### 4.5. Algorithms for Evaluating the Stability of Candidate Reference Genes

A total of four algorithms were adopted in this study to assess the stability of the oat reference genes: those of the geNorm [51], NormFinder [52], BestKeeper [9], and RefFinder [53] software programs. geNorm used the cycle threshold (Ct) values generated from qRT–PCR to calculate 2^−ΔΔCt^ as the treatment, and NormFinder and BestKeeper used the Ct values generated by the candidate reference genes from qRT–PCR as the treatment. In the geNorm analysis, the variation (V_n_/V_n + 1_) in the ratio of expression levels of n and n + 1 (n ≥ 2) reference genes were analyzed to calculate their standard deviation (SD), and the mean of SD was recorded as the average expression stability (M), which was used to indicate the stability of candidate reference genes. The smaller the M value is, the higher the stability of the reference genes. The NormFinder model is based on the ANOVA algorithm; the stability value (SV) is obtained by calculating the range of variation of the inner and outer clusters, and the smaller the SV is, the higher the stability of candidate reference genes. In BestKeeper analysis, the stability of the expression levels of reference genes is evaluated using both the coefficient of variation (CV) and SD; the smaller the CV value is, the greater the stability. Finally, RefFinder (http://blooge.cn/RefFinder/, accessed on 15 March 2022) was used for the comprehensive evaluation of suitable reference genes, which is a comprehensive analysis tool for evaluating gene expression stability when the results of geNorm, NormFinder and BestKeeper were inconsistent [54,55].

### 4.6. Validation of Selected Candidate Reference Genes

In order to verified the reliability of the results, the most stably and least stably expressed reference genes from the stability assessment results, and their combination were selected to normalize the expression levels of *PAL* [18] in the roots, stems, and leaves of oat under UV-B and highlight stresses with qRT–PCR analysis as described above, and the results were calculated using the 2^−ΔΔCt^ method. The experiment was conducted in three biological repetitions, and each individual run was repeated in technical triplicate.

## 5. Conclusions

In this study, eight candidate reference genes for qRT–PCR normalization under UV-B and high-light stresses in oat roots, stems, and leaves were systematically validated by the use of three algorithms, and the final comprehensive ranking was conducted by RefFinder. Based on the results of the present work, we recommend *PSKS1* as a suitable reference gene for all samples used for qRT–PCR assays, and the other genes can also be selected according to tissue and treatment specificity. The results of this study will help ensure the accuracy of normalization for qRT–PCR analysis in further molecular studies of oat under UV-B and high-light stresses.

## Figures and Tables

**Table 1:** Details of the primer sequences of candidate reference genes used in qRT–PCR analysis.

Gene Symbol	Gene Name	Primers (Forward/Reverse, 5′-3′)	Tm (°C)	Amplicon Length (bp)	qRT–PCR Efficiency (%)	R²
*SUOX*	sulfite oxidase	GAGCCTCATCGATCTGCGTT	80.08	132	103.42	0.9935
		ATTCACGAGGCCACTGATGG				
*VBP*	victorin binding protein	CCGTTACCTGCACAAGTTGC	81.80	117	109.03	0.9971
		GGATCGGTGACAGGCATCAT				
*Actin1*	actin-encoding	GGTATGGTCAAGGCCGGATT	87.07	111	101.53	0.9993
		ATCCTTCTGTCCCATCCCGA				
*PSKS1*	protein PSK SIMULATOR 1-like	TGGAGAGTGGGCAAATACAGG	81.06	136	92.61	0.9998
		AAGCCACACAACAAGCTCAAG				
*TBP2*	TATA-binding protein 2-like	GTGGATCTGTTGAGGCACCC	82.88	123	94.26	0.9930
		ATACTCTGCATTGCGCGCTT				
*UBC2*	ubiquitin-conjugating enzyme E2	ACAGCGTTCCGAGAGTTGTT	82.51	104	109.42	0.9629
		AAGAGTCAGCCCGAATGCAG				
*EF1-α*	elongation factor 1-alpha	AGCCTGGTATGGTTGTGACC	84.73	147	109.21	0.9947
		GCTTGAGATCCTTCACGGCA				
*GAPDH1*	glyceraldehyde-3-phosphate dehydrogenase 1	TTCTTCCTGAGTTGAACGGC	82.17	102	98.14	0.9970
		ATGCAGCCTTCTCGATTCTG				

**Table 2:** Expression stability of eight candidate reference genes under UV-B and high-light stresses according to the BestKeeper analysis results.

Rank	UV-B			High-Light			All Samples
	Roots	Stems	Leaves	Roots	Stems	Leaves	
1	*SUOX* (0.33)	*UBC2* (0.31)	*SUOX* (0.39)	*PSKS1* (0.37)	*UBC2* (0.37)	*EF1-α* (0.47)	*UBC2* (0.67)
2	*TBP2* (0.36)	*PSKS1* (0.58)	*UBC2* (0.51)	*UBC2* (0.41)	*SUOX* (0.38)	*PSKS1* (0.49)	*PSKS1* (0.67)
3	*PSKS1* (0.36)	*SUOX* (0.61)	*TBP2* (0.58)	*EF1-α* (0.46)	*TBP2* (0.52)	*TBP2* (0.54)	*TBP2* (0.74)
4	*GAPDH1* (0.43)	*GAPDH1* (0.63)	*VBP* (0.58)	*Actin1* (0.50)	*EF1-α* (0.55)	*Actin1* (0.58)	*SUOX* (0.96)
5	*VBP* (0.49)	*TBP2* (0.67)	*PSKS1* (0.59)	*TBP2* (0.53)	*GAPDH1* (0.58)	*GAPDH1* (0.66)	*EF1-α* (1.09)
6	*UBC2* (0.49)	*VBP* (0.86)	*Actin1* (0.83)	*GAPDH1* (0.60)	*PSKS1* (0.62)	*UBC2* (0.84)	*GAPDH1* (1.16)
7	*EF1-α* (0.50)	*EF1-α* (0.93)	*EF1-α* (1.09)	*VBP* (0.74)	*VBP* (0.65)	*VBP* (0.94)	*VBP* (1.69)
8	*Actin1* (0.64)	*Actin1* (1.03)	*GAPDH1* (2.02)	*SUOX* (0.81)	*Actin1* (0.78)	*SUOX* (0.98)	*Actin1* (2.28)

**Table 3:** Expression stability of eight reference genes under UV-B and high-light stresses according to the NormFinder analysis results.

Rank	UV-B			High-Light			All Samples
	Roots	Stems	Leaves	Roots	Stems	Leaves	
1	*EF1-α* (0.26)	*TBP2* (0.23)	*VBP* (0.09)	*PSKS1* (0.25)	*UBC2* (0.28)	*PSKS1* (0.17)	*PSKS1* (0.31)
2	*VBP* (0.29)	*GAPDH1* (0.27)	*UBC2* (0.35)	*TBP2* (0.27)	*GAPDH1* (0.38)	*EF1-α* (0.38)	*EF1-α* (0.47)
3	*SUOX* (0.41)	*SUOX* (0.34)	*PSKS1* (0.4)	*EF1-α* (0.32)	*TBP2* (0.38)	*TBP2* (0.39)	*TBP2* (0.71)
4	*TBP2* (0.45)	*PSKS1* (0.48)	*TBP2* (0.44)	*UBC2* (0.42)	*EF1-α* (0.39)	*GAPDH1* (0.43)	*UBC2* (0.83)
5	*GAPDH1* (0.55)	*VBP* (0.49)	*SUOX* (0.46)	*GAPDH1* (0.49)	*SUOX* (0.40)	*UBC2* (0.66)	*SUOX* (1.31)
6	*Actin1* (0.58)	*UBC2* (0.56)	*Actin1* (0.78)	*VBP* (0.52)	*VBP* (0.42)	*Actin1* (0.80)	*GAPDH1* (1.35)
7	*PSKS1* (0.61)	*EF1-α* (0.56)	*EF1-α* (0.81)	*Actin1* (0.72)	*PSKS1* (0.42)	*VBP* (0.81)	*Actin1* (2.27)
8	*UBC2* (0.62)	*Actin1* (0.71)	*GAPDH1* (2.73)	*SUOX* (0.85)	*Actin1* (0.65)	*SUOX* (1.30)	*VBP* (2.40)

**Table 4:** Comprehensive stability rankings of eight candidate reference genes.

Rank	UV-B			High-Light			All Samples
	Roots	Stems	Leaves	Roots	Stems	Leaves	
1	*EF1-α* (1.63)	*TBP2* (1.5)	*PSKS1* (1.97)	*PSKS1* (1)	*UBC2* (1.32)	*PSKS1* (1.19)	*PSKS1* (1.19)
2	*VBP* (2.11)	*GAPDH1* (2)	*VBP* (2.21)	*TBP2* (2.11)	*GAPDH1* (3.16)	*EF1-α* (1.41)	*UBC2* (2)
3	*SUOX* (2.45)	*SUOX* (3)	*TBP2* (2.45)	*UBC2* (3.13)	*SUOX* (3.56)	*TBP2* (3)	*EF1-α* (2.99)
4	*TBP2* (3.56)	*UBC2* (3.46)	*UBC2* (2.99)	*EF1-α* (3.22)	*TBP2* (3.57)	*GAPDH1* (4.23)	*TBP2* (3)
5	*Actin1* (5.18)	*PSKS1* (3.56)	*SUOX* (3.16)	*GAPDH1* (5.23)	*VBP* (3.98)	*Actin1* (5.18)	*SUOX* (4.73)
6	*GAPDH1* (5.18)	*VBP* (5.48)	*Actin1* (6)	*Actin1* (6.09)	*PSKS1* (4.14)	*UBC2* (5.48)	*GAPDH1* (6)
7	*PSKS1* (5.66)	*EF1-α* (7)	*EF1-α* (7)	*VBP* (6.24)	*EF1-α* (4.87)	*VBP* (7)	*Actin1* (7.24)
8	*UBC2* (7.44)	*Actin1* (8)	*GAPDH1* (8)	*SUOX* (8)	*Actin1* (8)	*SUOX* (8)	*VBP* (7.74)

## Data Availability

Not applicable.

## Supplementary Materials

The supporting information can be downloaded at: https://www.mdpi.com/article/10.3390/ijms231911187/s1.
