# Supplementary material for: Validation of Appropriate Reference Genes for qRT–PCR Normalization in Oat (Avena sativa L.) under UV-B and High-Light Stresses
Source: Int J Mol Sci. 2022 Sep 23;23(19):11187. doi: 10.3390/ijms231911187 (PMC9570368; doi:10.3390/ijms231911187)
Supplement: Supplementary file 1 [file ijms-23-11187-s001.zip › ijms-1897279-supplementary.pdf]

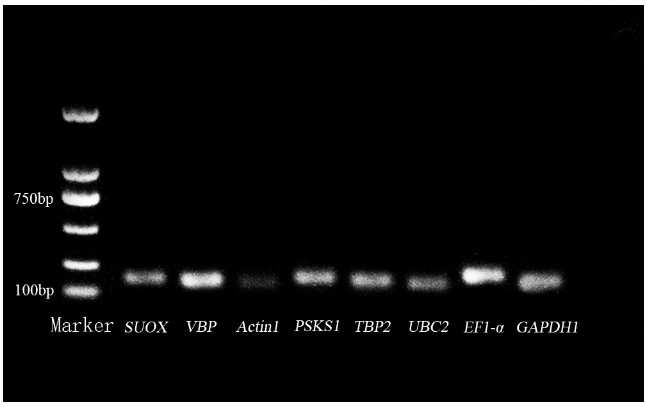

Figure S1: PCR amplified products of eight candidate reference genes. Marker: DL2000;

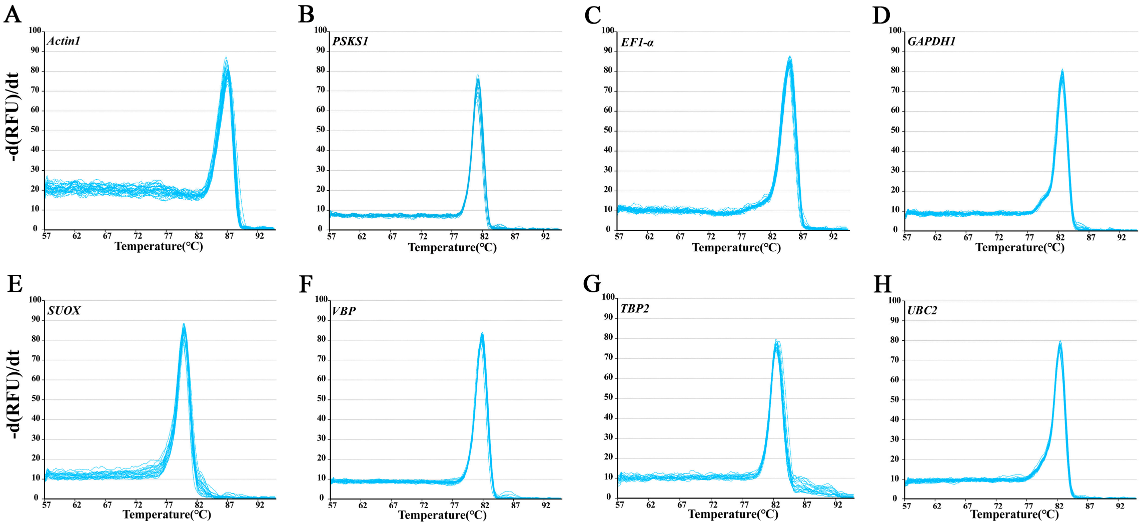

Figure S2: Melting curve analysis of eight candidate reference genes.
